# Human–wildlife conflict in the surrounding districts of Alage College, Central Rift Valley of Ethiopia

**DOI:** 10.1002/ece3.8591

**Published:** 2022-02-07

**Authors:** Zelalem Temesgen, Girma Mengesha, Tefera B. Endalamaw

**Affiliations:** ^1^ Ethiopian Biodiversity Institute Addis Ababa Ethiopia; ^2^ Wondo Genet College of Forestry and Natural Resources Shashamanne Ethiopia

**Keywords:** alage college, attitude, crop raiding, human‐dominated landscapes, livestock predation

## Abstract

The study was conducted between September 2018 and March 2019 to investigate the nature and extent of human–wildlife conflict (HWC) in the surrounding area of Alage College, the Central Rift Valley of Ethiopia, and to assess the perception of the local people to wildlife. For data collection, a total of 140 household (HH) heads were selected randomly for interviews from nine villages using structured and semi‐structured questionnaires. Moreover, focus group discussion, key informant interviews, and personal observation were carried out to obtain additional information. Descriptive statistics, Chi‐square test (2 tailed), one‐way analysis of variance, Pearson correlation coefficient, and Likert scale statements were used to analyze the data. Based on, 66 (47.1%) of the respondents, HH heads, the local people of the area experienced livestock predation leading to HWC. Whereas, (40.7%, *n* = 57) of the respondents perceived both crop damage and livestock predation as a cause of conflict. A total of 932.43 total livestock unit of livestock and 218 dogs’ losses were reported by HH due to predators over the last 5 years. Thus, the largest number of livestock (89.9%) and dogs (100%) attacks was happened due to spotted hyenas. *Nearly half of the respondents (49*.*3%*, *n* = *69) ranked warthogs as the primary crop raiders*, *while the majority of respondents (82*.*1%*, *n* = *115) reported maize as a severely damaged cereal crop*. More than half, (57.1%, *n* = 80) of respondents used different methods simultaneously to minimize damage caused by wild animals. About half, (48.6%, *n* = 68) of respondents had a negative attitude toward wildlife conservation. The level of education and amount of money imposed as a penalty for illegal grazing were affecting the local community's attitudes to wildlife conservation. Using effective methods to reduce damage and loss to crops, including improved livestock husbandry and creating better awareness to the local community could make the locals actor of conservation.

## Graphical abstract

Livestock depredation, crop‐raiding, and human attack conversely retaliatory killing of wild animals are the major manifestation of human–wildlife conflict in the present study. Hyena and warthog were reported as the most problematic livestock predators and crop raiders, respectively.

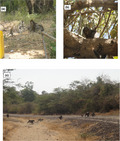

## 1 INTRODUCTION

The existence of human–wildlife conflict (HWC) goes back to time immemorial (Amare, 2015; Anand & Radhakrishna, 2017). HWC occurs when the needs and behaviors’ of wildlife affected human life negatively and vice versa (Yihune et al., 2009). Currently, it is a widespread phenomenon and challenge facing conservationists around the world (Acha & Temesgen, 2015; Mekonen, 2020; Mekuyie, 2014). It is a serious problem to those whose livelihoods are dependent on agriculture and livestock production (Girmay & Teshome, 2017; Kumssa & Bekele, 2014; Teshome et al., 2017), and those peoples living in and nearby wildlife habitats (Gebeyehu & Bekele, 2009; Tufa et al., 2018). HWC impacts range from crop‐raiding to livestock predation and human attack to other intangible social costs (FAO, 2015).

The rapid growth of the human population in developing countries and the requirement for more land for settlement and agriculture have lead to loss, degradation, and fragmentation of habitats inhabited by wild animals resulted in HWC (Acha & Temesgen, 2015; Berihun et al.,^2016^). The wild animals involved in HWC range from smaller (red locusts), non‐human primates to large herbivores. They cause vast damage to local people's crops and properties, and large mammalian carnivores cause livestock depredation and a threat to human life (FAO, 2010; Tufa et al., 2018).

Most of the Ethiopian wild animal resources have been restricted to protected areas due to a dramatic loss in natural habitat or coverage over the last few decades (Berihun et al., 2016; Ketema, 2017). This is particularly difficult for large carnivores which required a wide home range (Lagendijk & Gusset, 2008). This forced wild animals to spend some part of their lifetime on human‐dominated landscapes that are highly vulnerable to anthropogenic activities (Watson, 2010). Proximity to wildlife areas often creates conflict between humans and wildlife due to competition for shared and limited resources (Acha & Temesgen, 2015). This has led to HWC which has become a major threat for rural people to secure their household (HH) livelihood requirements (Mekuyie, 2014). As result, local people develop a negative feeling toward wildlife (Lagendijk & Gusset, 2008). This negative impact could have led to the clearing of vegetation to reduce the nuisance of wild animals and people stand antagonistic to wildlife conservation (Mojo et al., 2014).

In the Central Rift Valley of Ethiopia in particular the low land parts, wild animal habitats have been burned mainly due to charcoal production to be sold in the market and generate income resulting severe deforestation (Biazen, 2014). The high deforestation has resulted in a scarcity of resources for wild animals to fulfill their requirement of survival and production (Amare, 2015). The major conflicts that happened in human‐dominated landscapes were due to the segregation of wild animals in their farmlands or settlement areas (Makindi et al., 2014). These results in retaliatory killing (Tufa et al., 2018) and aggravating the disappearance of wildlife inhabited in human‐dominated areas (Masanja, 2014). A similar situation may happen in Alage, in the present study area where predators like; Spotted hyena (*Crocuta crocuta*) and Common jackals (*Canus aureus*), and crop raiders like; Warthog (*Phacochoerus africanus*), Olive baboon (*Papio anubis*), and Vervet monkey (*Chlorocebus pygerythrus*) were frequently seen (Derebe & Girma, 2020; Personal observation). However, there is no sufficient systematically studied information regarding HWC in the Alage area. Therefore, in this study, we investigated the impacts of HWCs on both humans and wildlife in the surrounding area of Alage. Specifically, we assessed (1) the types and extent of HWCs, (2) the main wild animals involved in HWCs, (3) the major driver causes of HWCs in the study area, and (4) people's attitudes toward wildlife conservation. Addressing these objectives will assist policymakers and conservationists in developing and implementing appropriate conservation policies that will aid in improving human–wildlife coexistence in human‐dominated landscapes.

## 2 MATERIALS AND METHODS

### 2.1 Description of the study area

**FIGURE 1:**
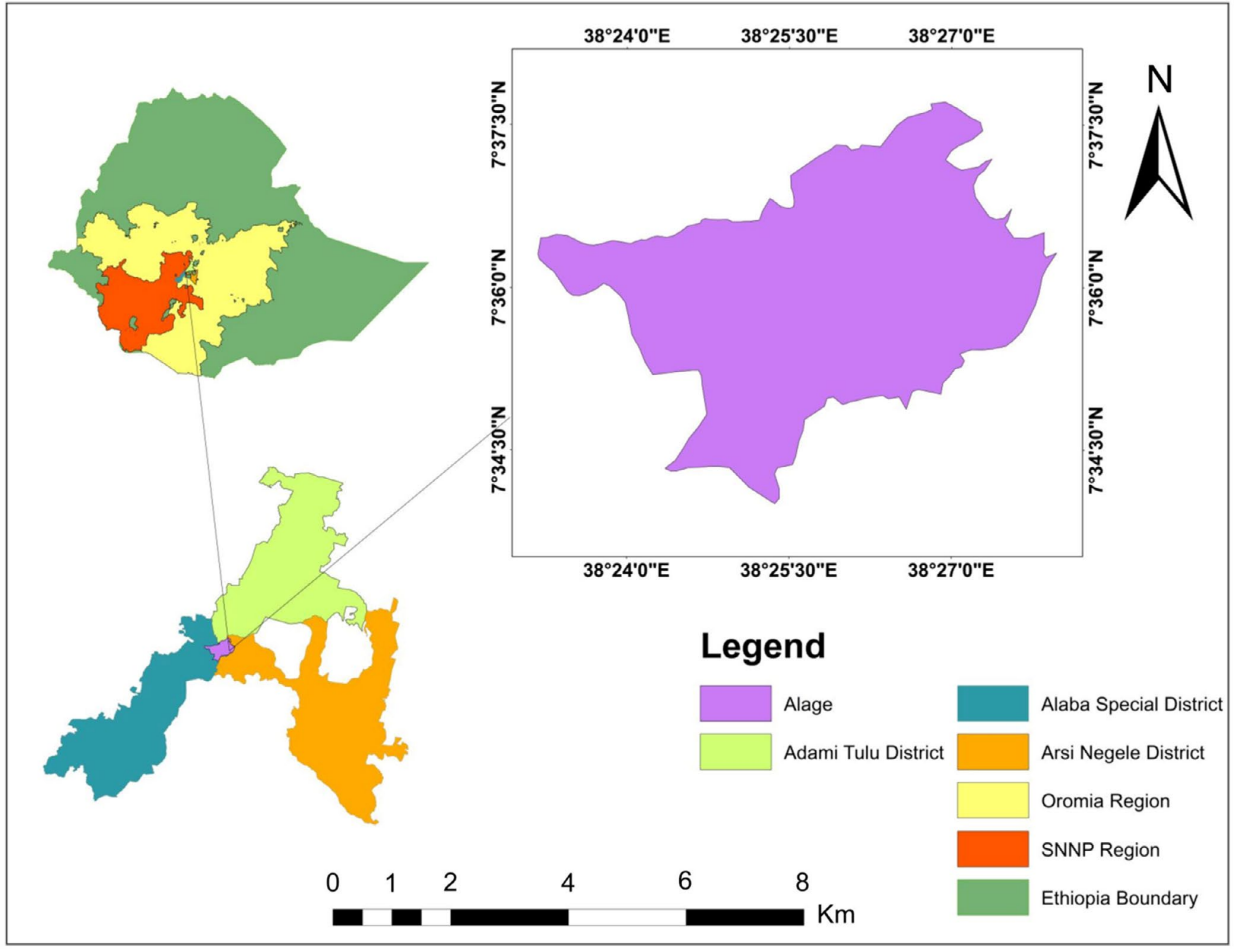
Map of Ethiopia showing the location of Alage College and its surrounding districts

**FIGURE 2:**
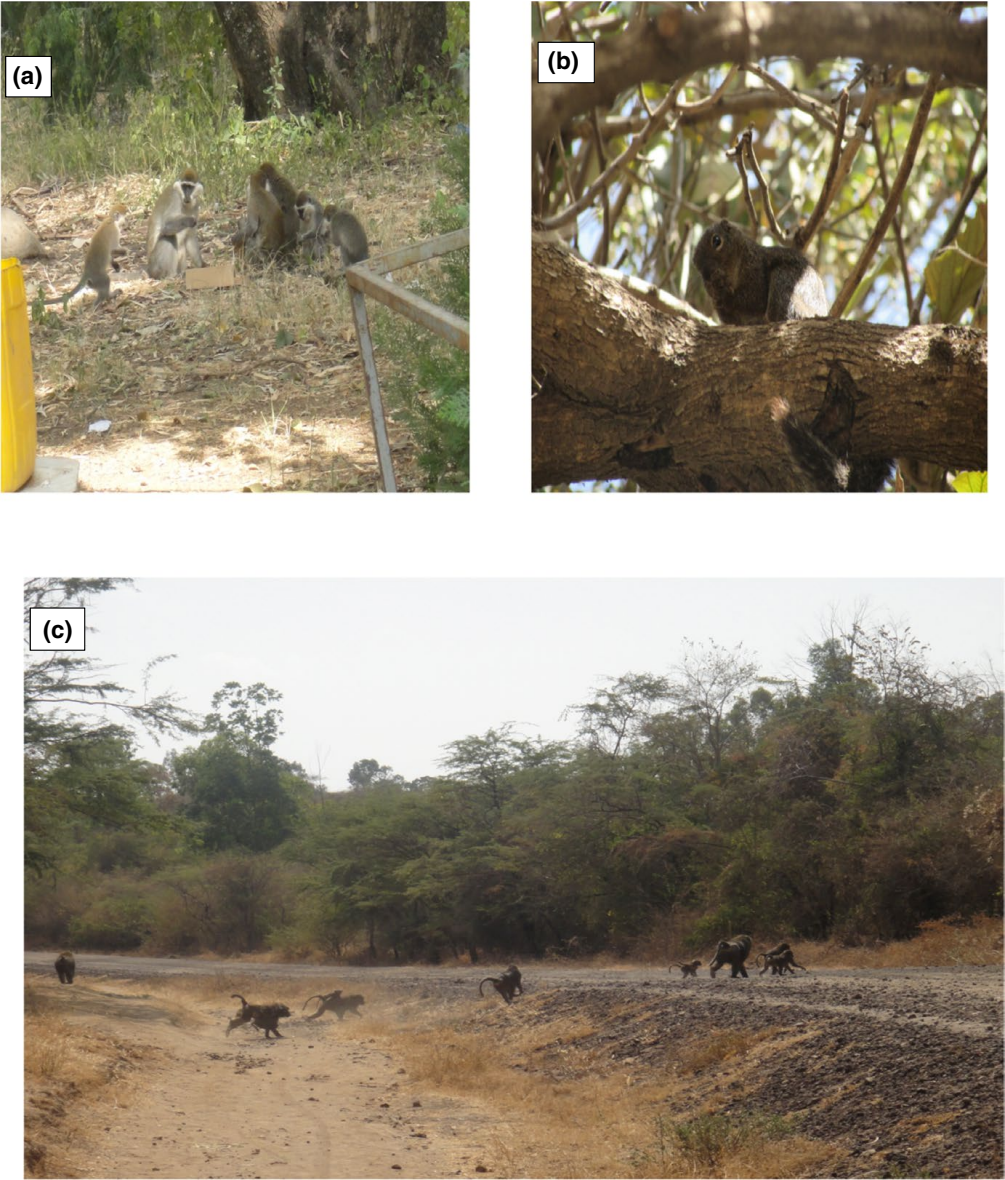
Some of the wild animals inhabited in Alage; (a) Vervet Monkeys around human settlement, (b) Squiller in riverial habitat, and (c) Olive baboons in Acacia wooded grassland

Alage is located in the Great East Africa Rift Valley, 217 km southwest of Addis Ababa and situated very close to *Abijata* and *Shala* Lakes, National Park, and west of Bulbula town at a distance of 32 km from Addis Ababa‐Shashemene highway. Alage shares its boundaries with Oromia Regional State (by Adami Tulu Judo Kombolicha and Arsi Negele districts) and SNNPRS (by Alaba special district). It is owned and managed by the Alage College management office. Geographically the study site is located in a range between 7°35′00″ and 7°37′30″N latitude and 38°25′00″ and 38°27′30″E longitudes (Figure 1). The area is characterized by bimodal rainfall distribution with average annual rainfall ranges from 700 to 900 mm and the average minimum and maximum temperature range between 6.8 and 34.5°C, respectively. It covers a total area of 29.46 km² lands with an altitudinal range from 1580 to 1650 ma bove sea levels. Its north, east, and northeast directions are bordered by the Jido River and the river served as the main water source of wild animals and livestock. The study area is dominantly covered by Acacia wooded grassland (55%) followed by opened grasslands (8.4%), and riverine and plantation forest (6.1%) (Derebe & Girma, 2020). Spotted hyena (*Crocuta crocuta*), Common jackals (*C. aureus*), Mongoose (*Atilax. paludinosus*), and Warthog (*P. africanus*), Olive baboon (*Papio anubis*), Vervet monkey (*C. pygerythrus*), Porcupine (*Hystrix cristata*), Antelope (*Gazella* spp.), and African civet (*Civettictis civetta*) (Figure 2) are the most commonly cited wild animals in the study area (Derebe & Girma, 2020; Personal observation). Both crop production and livestock rearing are the major economic activities of the communities (Biazen, 2014; Shiferaw et al., 2016; Tilahun et al., 2016).

### 2.2 Sampling design and data collection

Prior to data collection, 2 days reconnaissance survey was held to identify the Kebeles (the smallest administrative unit) with the highest incidences of HWC in the study area. Thus, a total of three Kebeles, namely; Alege‐Gero Kebele (from Alaba special district), Naka Kebele (from Adami Tulu Jido Kombolcha district), and Alge‐Delbtu Kebele (from Arsi Negele district) were selected purposively based on the highest incidences of HWC and proximity to the study area. Then, villages from each Kebele were stratified based on the distances category of near (<1 km), medium (1–5 km), and far (>5 km) from the study area. Following this, one village from each distance category was randomly selected. Therefore, a total of nine villages (three from each Kebele) were selected based on distance categories (near, medium, and far) of Alage. Accordingly, Boraa, Mansalega, and Rogedia villages were selected from Alege‐Delbtu kebele, from Naka kebele; Naka, Giro and Halaqee, and from Alege‐Gero kebele; Gotu, Machefar, and Huletegna‐Gotu villages were selected with estimated distances of near, medium, and far of Alage boundary, respectively followed by Nibret et al. (2017). After getting the total number of HHs living in each study Kebele from the population and housing census of Ethiopia (2007), a total of 140 HHs were selected for the questionnaire interviews based on a simplified formula developed by Yamane (1967) and reviewed by Israel (2012) with precision levels (e) of 8%. The sample sizes in each study Kebele were determined based on their proportion to the total HH of the three study Kebeles.

Then structured and semi‐structured questionnaires were used to explore the types and extent of HWC, the socioeconomic characteristic of the HH and resources use dependency, main wild animals involved in HWC, causes of HWC, mitigation measures, and the perceptions of local people to wildlife conservation. The questionnaires consisted of pre‐tested closed and open‐ended questions. The questions were also translated to the local languages (*Afaan Oromo* for Arsi ethnic group and *Alabigna* for Alaba ethnic group respondents), and the answers of the respondents were translated back to English. To gain people's attention and confidence as well as good information, the interviews were held in respondents’ homes (Holmern et al., 2004) whose age is ≥18 years old. Each respondent of the study villages was selected randomly by following a pattern of skipping two HH, and the third HH was interviewed (Mekuyie, 2014). To reduce exaggerating response, prior to each interview, the respondent was informed that the survey was independent of the government and that no compensation would be paid for any damage. We also assured them to use their information for research purposes, and that the study would be free of any political or religious concerns.

Three focus group discussants consisting of 8 to 12 participants and conducted after interviews to gather qualitative information about how local people perceived the interaction between humans and wildlife in the study area and their willingness to involve in future wildlife conservation. Participants of FGDs were selected based on years of residence and responsibilities (religion leaders and community leaders), and both sexes have participated. FGDs were held under the guidance of six well‐trained mediators; who are familiar with the local languages.

A total of, 12 Key informants were selected purposively (10 farmers and 2 wardens of the College). All questions for key informants were open‐ended. This was deliberately done to let the interviewees talk much about what they knew about human–wildlife interaction in the area.

### 2.3 Data analysis

The data were analyzed using Statistical Package for Social Science (SPSS) software version‐16. Chi‐square test was used to compute the differences between types of conflict and wild animals involved, crop damage by wild pests, causes of conflict, mitigation measures, livestock grazing, and firewood collection along with surveyed villages. One‐way ANOVA analysis of variance was applied to compute the mean differences of land owned, loss of livestock, and money paid as a penalty. Likewise, the Pearson correlation coefficient was used to test the relation between both the duration of grazing and firewood collection with distances and amount of money paid as a penalty with the duration of grazing, total livestock owned, and distances of villages from the study area. Mean values, ranges, percentages, and frequencies are also computed using descriptive statistics.

The attitudinal data contained 10 Likert Scale statements that were used to test the attitude of local people toward wildlife conservation. Each respondent responded to the 10 statements based on the five‐point Likert Scale method ranging from Strongly Disagree to Strongly Agree. Simple weightings (1–5) were assigned to the response categories. The maximum weight of 5 was given for ‘Strongly agree’ and the minimum 1 was assigned for ‘Strongly Disagree’. A weight of 2, 3, and 4 were given for the response categories of Disagree, Neither Agree nor Disagree (Neutral), and Agree, respectively. Thus, if a respondent gives 5 for all 10 statements, the maximum weight would be 50 whereas 10 would be the minimum weight when a respondent scores 1 for all 10 statements. Hence, the average of the sum scores of all 10 statements for each respondent would again range from 1 to 5. This gives another distribution of the data in which the mean and standard deviation is used to differentiate respondents according to their level of attitude toward wildlife conservation following the procedure applied by Gebrelibanos and Assen (2013). Higher average scores for statements indicate respondents had a positive attitude while lower scores show a negative attitude. The value of the Cronbach's alpha reliability coefficient of the present study of the Likert scale statements was 0.837 which indicates good internal consistency of the Likert Scale statements.

## 3 RESULTS

### 3.1 Socioeconomic characteristics of the respondents

The majority, 108 (77.1%) of the respondents’ HH earned their annual sources of income from both livestock and crop productions. While the remaining, 25 (17.9%) obtain their income from forest products, and trading 6 (4.3%) that served as supplementary income sources of HHs. The average landholding size (both farm and grazing lands) of the HH in the area was 2.44 ha with a maximum and minimum landholding size of 6 ha and landless, respectively. There was no significant (χ^2^ = 5.343, *p* = .069) difference in landholding size along with the study villages. The overall average numbers of livestock holding per HH were 19.564 ± 1.119 total livestock unit (TLU) with a minimum of 2.72 TLU and a maximum of 76.6 TLU numbers of livestock per HH, while the average number of dogs holding per HH was 1.89 ± 0.18 with a range between 0 and 8 dogs in a HH.

### 3.2 Nature and extent of HWCs

Nearly half of, 66 (47.1%) respondents HH reported that livestock predation was a major cause of HWC, while 57 (40.7%) HH perceived both crop damage and livestock predation problems in the present study. Few, 13 (9.3%) respondents HH reported that it was not a problem. Type of conflict the community experienced were statistically significant (χ^2^ = 101.287, *df* = 8, *p* = .043) along the surveyed villages.

**FIGURE 3:**
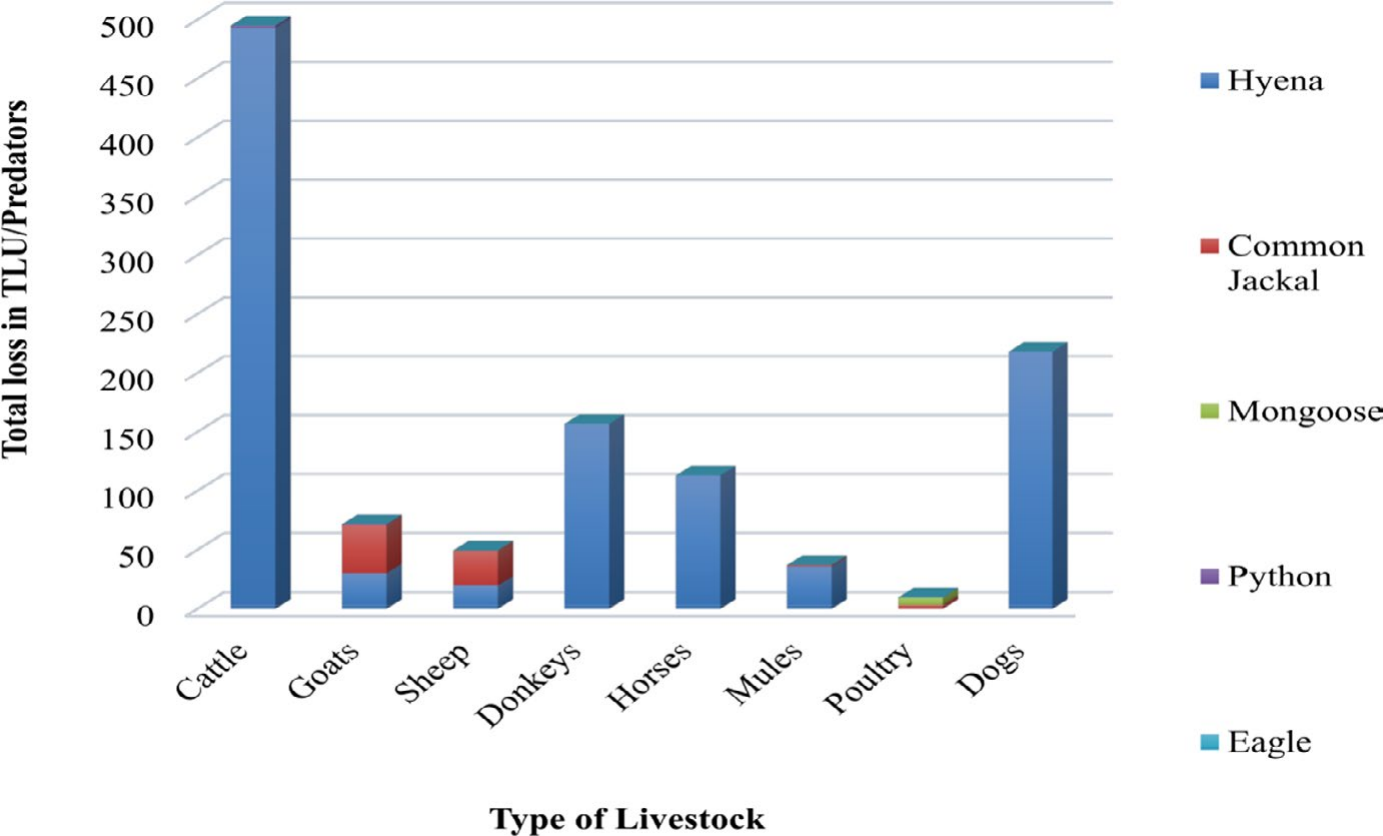
Total number of domestic animals loss per predators over the last 5 years as reported by the respondents

Respondents also viewed the type of predators that killed livestock. According to respondents, Hyena and Common jackal were the main livestock predators in the present study (Figure 3). In total, 932.43TLU livestock and 218 dogs’ predation incidences were reported by survey respondents over the last 5 years. Thus, cattle (*Bos taurus*) (495 LTU) were predominantly predated followed by dogs (218), whereas the least predation incident was reported on poultry 9.63 LTU. Hyena was responsible for all cattle, dogs, donkeys, horses, and mules’ losses (Figure 3). On average 6.376 ± 0.625 TLU livestock and 1.343 ± 0.253 numbers of dogs’ loss per HH reported in the last 5 years. The frequency of livestock predation was significantly different among the surveyed villages (*F* = 8.157, *df* = 8, *p* = .001). However, distance from the edge of Alage had no significant impact (χ^2^ = 8.695, *df* = 8, *p* = .74) on the livestock predation risk in the present study (Table 1). For example, the highest predation incidents were reported from Giro and Halaqee villages within distances category of medium (1–5 km) and far (>5 km), respectively, while the least predation incidents were reported from Machefar and Huletegna Gotu villages within distances category of medium (1–5 km) and far (>5 km), respectively.

**TABLE 1:** Number of domestic animals depredated per village in the last 5 years as reported by the respondents

Villages	Number of livestock and dog depredated in the last 5 years							
	C	G	SH	Do	H	M	P	D
Boraa (<1 km)	115	11.8	6.5	23.1	24	7	0.32	3^a^
Mansalega (1–5)	77	15.2	9.5	25.9	17	7	1.84	–
Rogedia (>5)	43	10.9	8.1	15.4	1	–	1.14	5^a^
Gotu (<1)	1	2.2	–	6.3	1	–	0.51	2^a^
Machefar (1–5)	1	1	0.3	–	–	–	0.18	–
Huletegna Gotu (>5)	4	1.2	0.2	–	–	–	0.04	–
Naka (<1)	48	5.2	5.2	20.3	29	9	1.16	45^a^
Giro (1–5)	100	11	9.4	33.6	23	8	2.71	92^a^
Halaqee (>5)	106	13.1	10.2	32.2	18	6	1.73	71^a^
Total losses	495	71.6	49.4	156.8	113	37	9.63	218^a^

Abbreviations: C, Cattle; D, Dog; DO, Donkeys; G, Goats; H, Horses; M, Mule; P, Poultry; SH, Sheep.

^a^ The number is not in TLU.

Regarding crop‐raiding, 69 (49.3%) respondents’ reported warthog as a topmost crop raider wild animal followed by Verevt monkey 31 (22.1%) (Table 2), whereas 115 (82.1%) respondents ranked maize (*Zea mays*) as a primary and most commonly raided crop (Table 3) in the study area. Types of wild animals involved (χ^2^ = 88.468, *df* = 8, *p* = .03) and crop damaged by pests animals (χ^2^ = 70.108, *df* = 8, *p* = .025) were significantly different among surveyed villages. Distances from Alage and crop damage events were negatively correlated (*r* = −.396, *p* = .005) in the present study.

**TABLE 2:** Major crop raider identified during the study (*N* = 140)

Crop raiders	Frequency	Percentage
Warthog (*Phacochoerus africanus*)	69	49.3
Vervet Monkey (*Chlorocebus pygerythrus*)	31	22.1
Olive Baboon (*Papio anubis*)	21	15
Porcupine (*Hystrix cristata*)	10	7.2
Others	9	6.4
Total	140	100

Note

Others (Antelope species, Squirrel, African Civet, Bird spp., and rodents).

**TABLE 3:** The most frequently raided crops by wild animal pests (*N* = 140)

Type of crops	Frequency	Percentage
Maize	115	82.1
Sorghum	12	8.6
Barley	7	5
Chile Paper	6	4.3
Total	140	100

### 3.3 Major driver to causes of human–wildlife conflict

**FIGURE 4:**
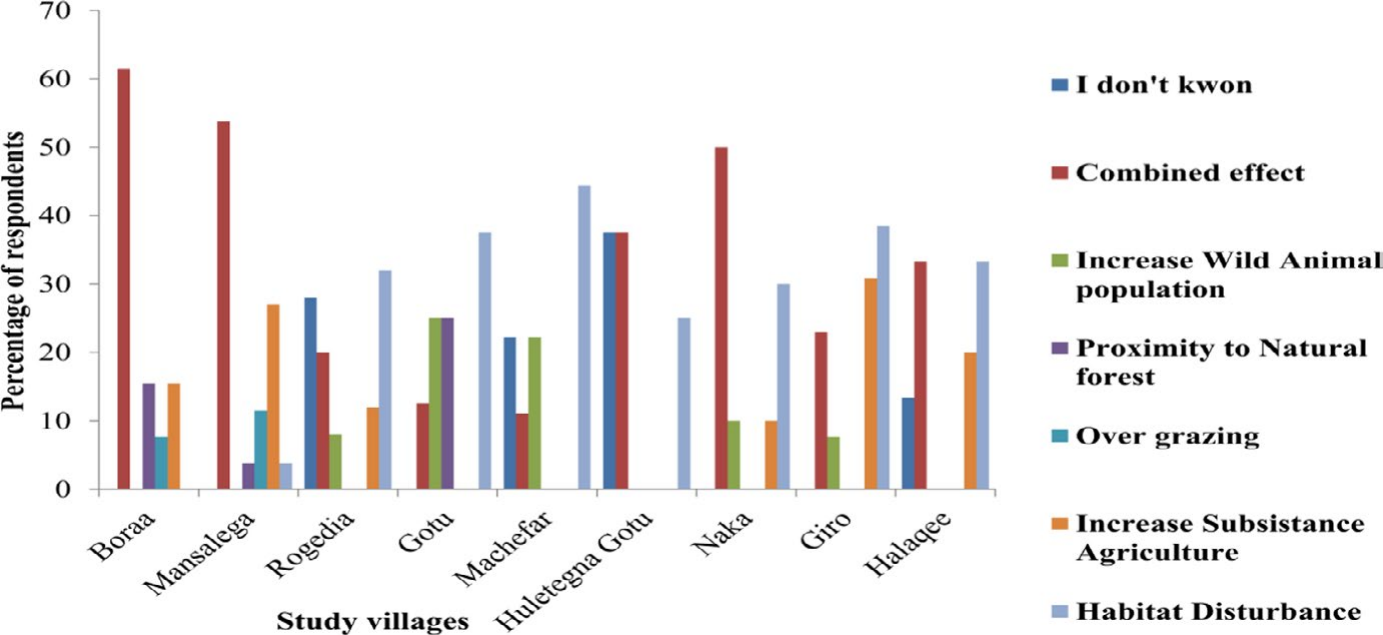
Respondents’ view of major drivers to cause of human–wildlife conflict (*N* = 140)

The majority, 53 (38%) of surveyed respondents thought that a major drivers cause of HWC were driven due to combined effects of anthropogenic activities. However, six percent of the respondents perceived the increasing wildlife population as a cause of HWC in the study area (Figure 4). The perception of respondents about the major driving forces of conflict was statistically significant (χ^2^ = 1,31,988, *df* = 8, *p* = .005) across the study villages.

### 3.4 Mitigation measures

More than half of, 80 (57.14%) respondents used guarding, chasing, fencing, smoking, and scarecrow simultaneously to minimize damage caused by wild animals in the study area (Table 4).

**TABLE 4:** Traditional methods the local people used to reduce wild animal damage (*N* = 140)

Type of Traditional methods	Frequency	Percentage
Guarding and chasing	38	27.14
Fencing, smoking, and scarecrow	2	1.43
Guarding, chasing, fencing, smoking, and scarecrow	80	57.14
Killing problematic wild animals	20	14.29
Total	140	100

**FIGURE 5:**
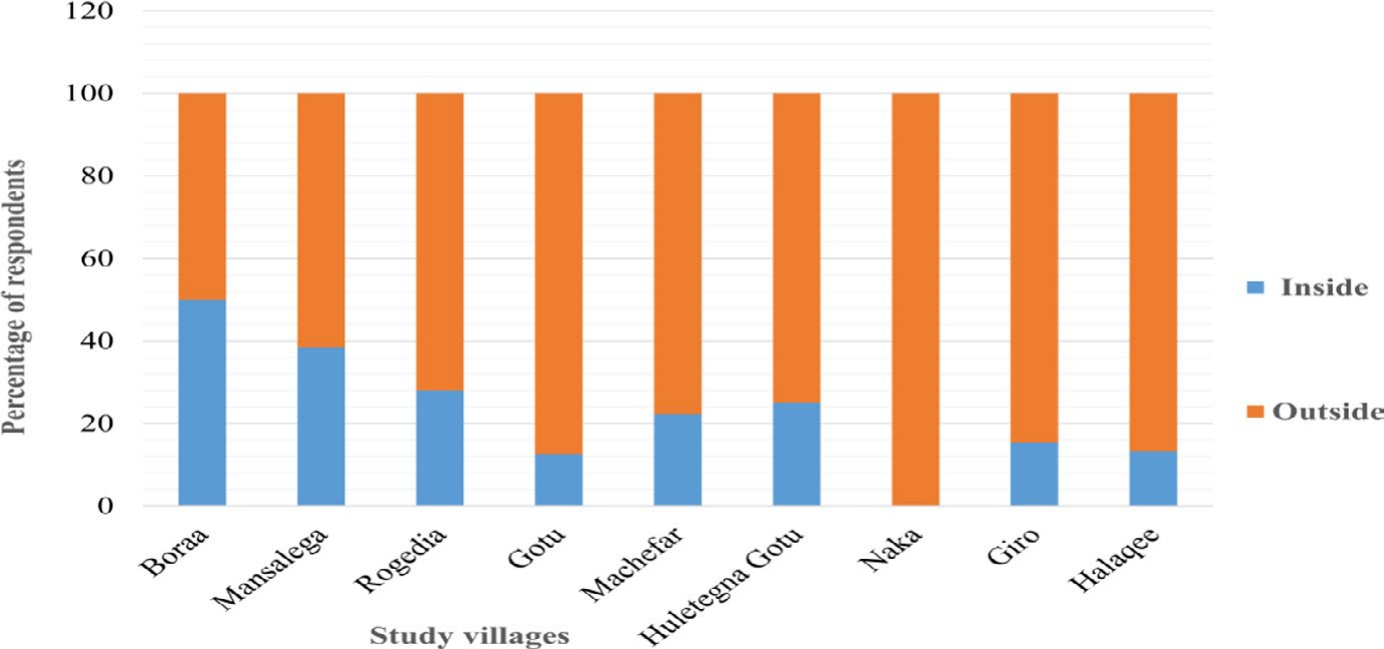
Way of keeping livestock during night‐time across the study villages (*N* = 140)

In the case of livestock husbandry, 101 (72.1%) of HHs kept their livestock outside the home overnight. Of the remainder, 27.9% kept their livestock inside traditional enclosures (Figure 5). The level of predation incident had no significant differences (χ^2^ = 6.963, *df* = 4, *p* = .069) between HH who kept their livestock inside and outside enclosures’ during night times.

### 3.5 Resource dependences and a penalty for illegal grazing

The local community used the natural resources as livestock grazing (46%) and as sources of firewood (46.4%) for their house (Table 5). livestock grazing (*χ* ^2^ = 108.955, *df* = 8, *p* < .001) and firewood collection (*χ* ^2^ = 83.452, *df* = 8, *p* < .001) were statistically significant among the surveyed villages. Both duration of grazing (*r* = −.552, *p* < .001) and firewood collection (*r* = −.705, *p* < .001) were negatively correlated along distance from the study area.

**TABLE 5:** Respondents’ perception of utilizing the forest for grazing and firewood among surveyed villages

Villages (estimated distance in km)	*N* = Number of respondents				
	Grazing inside the forest			Firewood collection from the forest	
	*N*	Yes (%)	No (%)	Yes (%)	No (%)
Boraa (<1 km)	26	100	0	100	0
Mansalega (1–5)	26	92	8	69	31
Rogedia (>5)	25	4	96	4	96
Gotu (<1)	8	62.5	37.5	87.5	12.5
Machefar (1–5)	9	0	100	11.1	88.9
Huletegna Gotu (>5)	8	0	100	0	100
Naka (<1)	10	70	30	70	30
Giro (1–5)	13	8	92	30.8	69.2
Halaqee (>5)	15	0	100	6.7	93.3
Total	140	46	54	46.4	53.6

**FIGURE 6:**
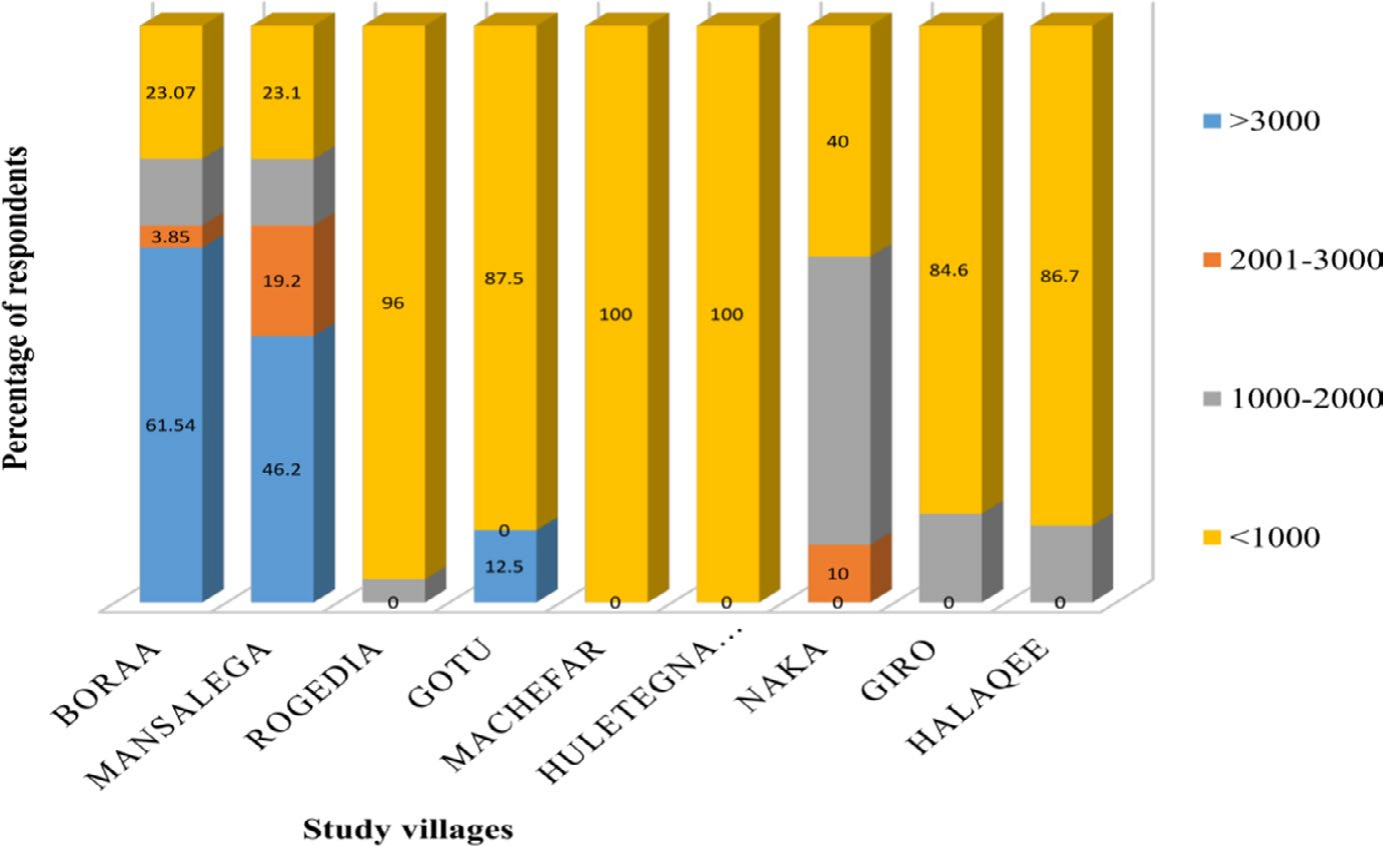
Respondents’ estimation of money paid as penalty due to illegal grazing per villages in the last 5 years (ETB; 1 US DOLLAR = 28.1089 ETB)

The average amount of money paid as a penalty per HH in the last 5 years in Ethiopian Birr (ETB) was 1,288.62 ± 153.07 (Figure 6). The total amount of money paid as penalty was positively correlated with both duration of grazing (*r* = .502, *p* < .001) and total livestock owned (*r* = .486, *p* = .005), while negatively correlated with distance (*r* = −.476, *p* = .01) along villages from the border of the study area.

### 3.6 Local people perception toward wildlife conservation

According to the present findings, a few (24.3%) of the respondents had a positive feeling while some (27.1%) respondents had a neutral feeling. On the contrary, nearly half (48.6%) of the respondents had an unfavorable feeling toward the conservation of wildlife (Table 6). Relatively uneducated people had high negative attitudes (χ^2^ = 37.585, *df* = 4, *p* < .001). Besides, people who paid a high penalties (χ^2^ = 20.888, *df* = 3, *p* < .001) would develop high negative attitudes toward co‐existence as compared to those who did not yet.

**TABLE 6:** Respondents’ perception toward Wildlife conservation across villages (*N* = 140)

Attitude	Frequency	Percentage
Positive	34	24.3
Neutral	38	27.1
Negative	39	27.9
Strong negative	29	20.7
Total	140	100

## 4 DISCUSSION

As shown by the current results and reported studies (e.g. Mekuyie, 2014; Shanko et al., 2021; Yilmato and Takele, 2019), local people and wildlife in human‐dominated landscapes are constantly in conflict. These mainly took the form of livestock depredation, crop‐raiding, and human threats, as well as the deliberate killing of wild animals in retaliation.

**FIGURE 7:**
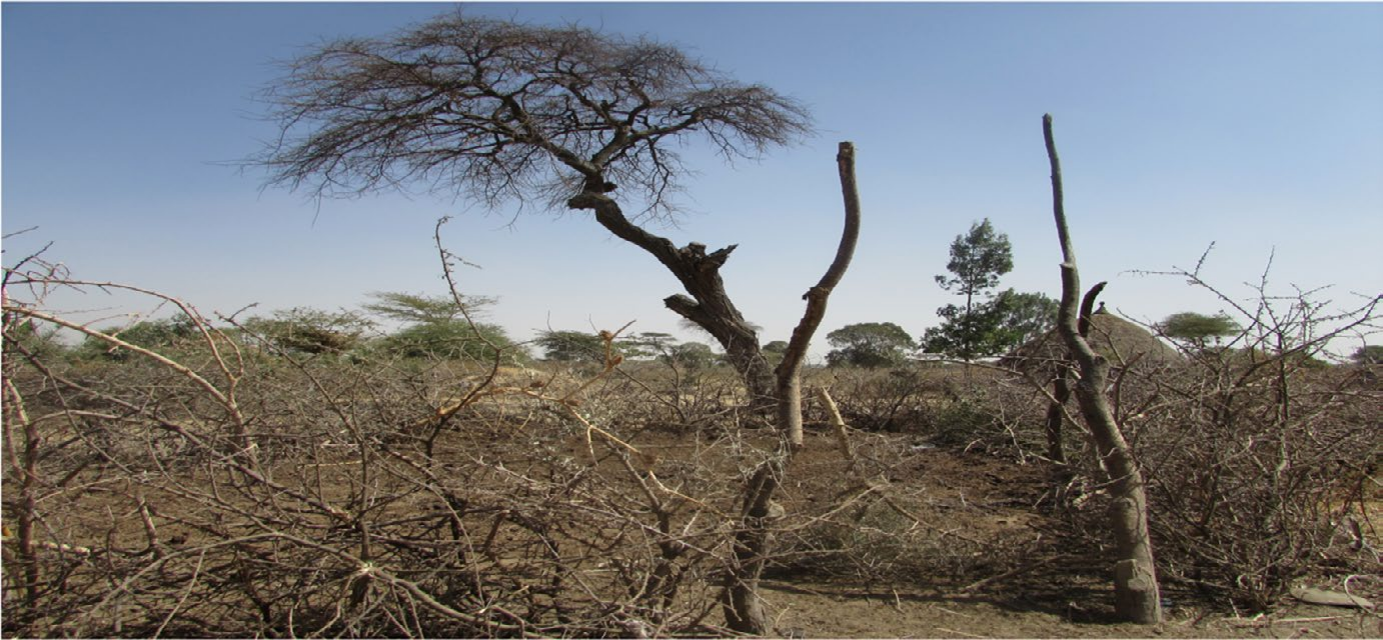
Type of traditional enclosure used to keep livestock during night

The spotted hyena and common jackal were the most common livestock predators reported in the present study. Similarly, both predators were identified as predominant livestock predators in Belo‐Bira Forest, Southwestern Ethiopia (Shanko et al., 2021), and in northern Ethiopia's highlands (Yirga et al., 2012). Mekonen (2020) also reported both hyenas and common jackal as the most predominant livestock and domestic dog predators in and around Bale Mountains National park. Distances from Alage and livestock predation were not related in the present study. This could be because hyenas and common jackals have highly adaptive behavior in human‐dominated areas (Tufa et al., 2018; Yirga et al., 2012). This is in line with the findings of Yihune et al. (2008), who found that distance from the park has no effect on sheep predation due to the presence of Ethiopian wolves outside of Simien National Park. In contrast, Nibret et al. (2017) revealed a negative correlation between livestock predation and distance from the forest. Moreover, poor animal husbandry practices (Figure 7) and Hyenas’ ability to kill all types of livestock from cattle to poultry, as well as a flock of sheep and goats at the same time may have resulted in substantial losses (932.43 TLU and 218 dogs). These were significant losses as compared to predation in the Arsi Mountains National Park (Tufa et al., 2018) and Chebera Churchura National Park (Datiko and Bekele, 2013), and equivalent to cattle depredation reported by Yirga et al. (2012) in northern Ethiopia's highlands.

Warthog, Olive Baboon, Vervet Monkey, Porcupine, and African Civet were also recognized as major crop pests elsewhere in Ethiopia (Gebeyehu & Bekele, 2009; Mekuyie, 2014; Girmay & Teshome, 2017; Tufa et al., 2018). Focus group participants and key informants also emphasized that Olive Baboon and Vervet Monkey are difficult to chase out of crops because of their sophisticated social organization habits and capacity to climb surrounding trees. The intensity and vulnerability of crops being damaged by wild animal pests were varied depending on the type of crop planted (Mekonen, 2020) and its land coverage, and the type of wild animal involved in crop‐raiding (Gobosho et al., 2015; Mc Guinness & Taylor, 2014). Farmlands in close proximity to wild animal habitats are known to be frequently visited by crop raiders and are more vulnerable to damage (Mamo et al., 2021; Nibret et al., 2017). Maize was the most preferred crop by crop raiders, followed by sorghum. The possible reasons could be due to its nutritional values (Raphela & Pillay, 2021), and large farm sizes in proportion to other crops in the area.

In the study area, the natural habitat of wild animals has recently been altered for livestock fattening and horticulture practices. The remaining fragmented habitats are unable to support wild animals for an extended period of time (personal observation). As a result, wild animals were more likely to be seen, resulting in HWC. Gobosho et al. (2016), Admassu et al. (2014), and Mekonen et al. (2017) found that deforestation, habitat loss, degradation, and agricultural land expansion were identified as the most serious threats to wild animals living in human‐dominated areas, as well as the leading sources of HWC.

Farmers in the study area used different methods simultaneously to protect their property from predators and crop raiders. Sometimes local people may kill wild animals in response to crop damage, livestock depredation, and threat to humans even if they knew it is illegal. Similarly, the local communities in Gera district, Southwestern Ethiopia used guarding, chasing, fencing, scarecrow, and smoking to reduce crop damage and livestock predation (Gobosho et al., 2015). Farmers in Kenya also used different crop protection methods simultaneously depending on the type of raiders involved (Musyoki, 2014). The need for controlled hunting of wild animals (warthogs, baboons, and monkeys) was perceived by the villagers as a management option for reducing crop damage. Retailer killing of large carnivores was used to reduce livestock‐carnivore conflicts elsewhere in the world (Gandiwa, 2011).

The local people send their livestock for searching fodder and water during severe drought although their dependence differs from village to village. As distances decreases, use increase. Thus, the livestock owners’ are exposed to penalties as a result of illegal grazing. The focus groups and key informants emphasized the need for allowing restricted livestock grazing during drought seasons. A similar finding was reported by Nibret et al. (2017) the communities living nearby Aba‐Jemie forest utilized the forest for both grazing and firewood throughout the year. The present finding is also in line with Gebeyehu and Bekele (2009) local communities living in Zegie Peninsula utilized the forest as firewood for house consumption and market sale as a means of alternative sources of income for their HH.

Restriction of access to wildlife resources, penalties as a result of illegal grazing and illiteracy can result in unfavorable perceptions toward wildlife conservation among local people in the present study. This is in line with the findings of Gezahagn et al. (2014) and Shi et al. (2010), who reported that restricting access to wildlife resources and enforcing punishments have a negative impact on the perception of the local people. Residents with formal education were more appreciated conservation objectives (Karanth & Nepal, 2012; Shibia, 2010). In contrast to the findings of Eshete et al. (2018), who found that even low levels of livestock predation might lead local people to develop negative attitudes toward wildlife, surprisingly, in the current study, a high level of predation has no significant negative impact on local people's perceptions. This could have significant implications for wildlife conservation in human‐dominated areas.

## 5 CONCLUSION AND RECOMMENDATIONS

As indicated by the current study, where subsistence farming is a major income source of the HH, high levels of conflict could occur between humans and wildlife. Therefore, losing their property and human threats due to wild animals may sometimes lead the local people to kill wild animals intentionally and develop unfavorable perceptions toward wild animals. Moreover, habitat destruction for subsistence farming, overgrazing, and proximity to wildlife habitat were the major factors. Thus, combined anthropogenic factors and to some extent increase in wildlife in the area escalated the conflict. Poor livestock husbandry was attributed to the loss of large numbers of livestock by spotted hyenas and common jackals in the study area. Conservation education needs to be given rather than imposing inappropriate penalties. Livestock husbandry needs to be improved and the livestock enclosure needs to be well built. Further study is important to understand the abundance and diversity of fauna and enhance the coexistence of humans and wildlife in the area.

## CONFLICT OF INTEREST

The authors declare that we have no competing interests.

## AUTHOR CONTRIBUTIONS

**Zelalem Temesgen:** Conceptualization (lead); Data curation (lead); Formal analysis (lead); Funding acquisition (lead); Investigation (lead); Methodology (lead); Project administration (lead); Resources (lead); Software (lead); Supervision (lead); Validation (lead); Visualization (lead); Writing – original draft (lead); Writing – review & editing (lead). **Girma Mengesha:** Methodology (supporting); Software (supporting); Supervision (supporting); Writing – review & editing (supporting). **Teferra B. Endalamaw:** Methodology (supporting); Software (supporting); Supervision (supporting); Writing – review & editing (supporting).

### OPEN RESEARCH BADGES

This article has been awarded Open Materials, Open Data, Preregistered Research Designs Badges. All materials and data are publicly accessible via the Open Science Framework at [https://datadryad.org/stash/share/tOjKfqJDMK6SGGOkuj0IOWyQDr084pNPCHu1xUi8YjQ].

## Data Availability

The data that support the findings of this study are publicly available at https://doi.org/10.5061/dryad.02v6wwq4z.
